# Student Ideals1Opening lecture of the Sixty-first Annual Course at the University of Buffalo, delivered September 24, 1906.

**Published:** 1906-11

**Authors:** James E. King

**Affiliations:** Buffalo, N. Y., Adjunct Professor of Obstetrics, University of Buffalo, Attending Gynecologist, Buffalo General and Erie Co. Hospital; Fellow British Gynecological Society


### BUFFALO MEDICAL JOURNAL.

VOL. LXII. NOVEMBER, 1906. No. 4

### ORIGINAL COMMUNICATIONS.

### STUDENT IDEALS.[1]

BY JAMES E. KING, M. D., Buffalo, N. Y.

Adjunct Professor of Obstetrics, University of Buffalo, Attending Gynecologist, Buffalo General and Erie Co. Hospital; Fellow British Gynecological Society.

I have always considered the one selected to give the opening lecture of the year, in the medical department of our University, as a particularly favored person. It is the only time that a member of the Faculty has the opportunity to meet members of the four classes, and is at the same time permitted to choose his own subject for discourse. I find myself in that enviable position tonight and I want to take the occasion to welcome you all back, to say what I can to you of encouragement, and above all to give you some good advice. It is human nature for all and a second nature for physicians to tell others what they ought to do. In those of our profession, therefore, there may be found an excuse for this weakness and behind that excuse I shall take refuge, if you consider that I am taking too great an advantage of you at this time.

We begin tonight the sixty-first year of our medical department. Sixty years of honorable record, during which time our university has sent out its share of medical men until now they may be found in almost every state of the union. Early in our history as well as in the history of all medical schools, it was an easy matter, comparatively, to obtain the degree which you now all are seeking. Two years of four to six months each was all that was required. A common school education was all that was necessary as preliminary and this enabled many a farmer to step out of his overalls into the profession of medicine. No doubt in this way many a good farmer was lost to the world in the making of a poor doctor. It is yearly becoming more difficult to enter upon the study of medicine. The preliminary requirements are continually being raised, the study course is from time to time

1. Opening lecture of the Sixty-first Annual Course at the University of Buffalo, delivered September 24, 1906.

lengthened and made more thorough, and it is a very safe prediction, that you will live to see the time when five years will be the shortest period in which the degree of doctor of medicine may be obtained. I say this for the consolation of the freshmen who may be here tonight, and who may perhaps consider that four years is a long, long time. Each year as it passes, however, will seem shorter than the preceding, until at last those four years will really have slipped by very quickly and you will be reverenced by the then freshmen as I suppose you reverence the seniors now.

I think that the freshmen should have all the encouragement and kinds words that we can spare. Such words of encouragement must come from the faculty, for the kinds words that the freshmen receive from the other classes, especially the sophomores, will never spoil them. There are three occasions in the life of every freshman which I am sure way down deep in his heart he dreads. They are his first official meeting with the sophomores, his first operation, and his first visit to the dissecting room. I have never quite understood why the duty and pleasure of welcoming the freshman class always fell upon the sophomores. They have always seemed, however, to have accepted the trust with pleasure and enthusiasm and to have discharged their duties with neatness and dispatch. The only explanation that I could ever give is that the sophomores being the last class to be welcomed, there remains fresh in their memories the details of their own experience during those interesting ceremonies, and this stimulates their inventive genius and causes them to enter into the spirit of the occasion with great zest. As to his first operation, there is always a question in his mind as to just how much of that operation he will see. On that occasion he is usually found in close juxtaposition to the emergency exit. Should he withstand the ordeal, however, he leaves the clinic with his chest a few inches higher, a thorough conviction of the nobleness of medicine in general and of surgery in particular, and a firm mental resolve that he himself will one day be a surgeon. If, on the other hand, he should be seized during the operation with a sudden and uncontrollable desire to lie down, surgery then does not possess for him the interest it ought, and after he has been propped up in his seat and the great beads of cold sweat stand out on his brow and trickle down his neck, he wonders if he has made a mistake in thinking that he is really needed in the ranks of the medical profession. This experience rarely happen the second time and in a few weeks our freshman is found in the front rows, absorbing with interest the details of the various operations.

The dissecting room, too, at first has for him a certain horrible charm. This feeling is, perhaps, encouraged by the attitude of the laity in general and the members of his family in particular. A medical student is supposed generally to revel in horrible things. He is viewed with suspicion abroad as well as at home, and all mysterious packages that he may carry or bring home are supposed to contain fragmentary portions of "the human form divine." The dissecting rooms today do not possess the many unpleasant features of years ago. The dissections were then done at night, which, of course, added much to the unpleasantness of the duty. The work, too, was conducted with much less thoroughness and system than at present. In my students days we were required to do a certain amount of dissection in a certain specified length of time. The record of time and work was kept in the dissecting room in a large book, in which we were required to register, indicating the time of our arrival and upon leaving the time of our departure, together with the particular amount of work done for the evening. This constituted the only official record of time spent in the dissecting room, and this rather loose system made it possible for those disposed to make false entries of time and work.

I am glad to say, however, that there were but few students who took advantage of this opportunity to be unfair. I remember on one occasion, however, one of the students came early to the dissecting room one winter's evening, being on his way to the theater. He came to register his name and so get credit for time not actually spent in the room. He was dressed in his best and it was rather exasperating for us, in our aprons, to see him standing about in his finery, smoking a cigarette while we were doing honest work. We all cheered up presently, however, when we saw one of the students surreptitiously slip into his overcoat pocket three long cold fingers, in an excellent state of preservation and whose days of usefulness were over. Our wildest hope was that later, perhaps, he would put his hand into his pocket and receive a mild shock and perhaps even a twinge of conscience at the reminder of his having falsely and dishonestly registered. What actually did happen we learned the following morning. It appears that our friend and his young lady reached the theater without mishap and he placed his overcoat in a vacant seat next his girl. During an affecting scene in the first act, the young lady requested the opera glasses and he whispered back that they were in his overcoat and that if she would feel in his pocket she would find them. She evidently did find something, for he said that the celerity with which that young woman's hand was withdrawn from that pocket was little short of marvelous. With a look of horror on her face she asked him how he could play such a mean trick on her. He, of course, was ignorant as to why he should have taken this sudden fall into disfavor, and by way of explanation he reached over into his pocket and brought to the surface the three fingers. The young lady promptly became hysterical and insisted on being taken home at once. The following evening he was seen going about the dissecting room with the three digital relics in an endeavor, with the instincts of a true Sherlock Holmes, to match them with a view to discovering the perpetrator of the trick. His efforts were, of course, without success. "Dead men tell no tales."

I think that it would be very interesting if a perfectly candid expression of opinion could be obtained from each student as to why he was prompted to adopt medicine as his profession. For yourself and your future it is very important that you know your true motive. Were you to so honestly analyse your motives I think they would be found to be among the following reasons: First, perhaps, you thought that the pecuniary reward from the practice of medicine will be great. If such is the case you are probably doomed to disappointment. Men actuated by this motive compose a large percentage of those who either give up the profession entirely, or who leave legitimate medicine for the ways of the charlatan. Again, you may have thought that the profession of medicine assured you a more honorable standing in your community than other pursuits, but I can assure you that in this democratic country of ours it is useless for you to depend upon your title of doctor alone to do you honor. It is only by your own worth as men and women and by your deeds as physicians that you may expect the title of doctor to bring you honor and standing among men. On the contrary, it has become a constant strife to raise and protect our profession from the hands of those who have the same degree as we, but who practice all kinds of irregular methods calculated to cure the sick.

Possibly, too, some one may have turned to medicine for the same reason that a rather discouraged father gave, when he explained that as his son had failed in everything else he had to make a doctor of him. Perhaps that was possible years ago, but medicine today is not the dumping ground for miserable failures. For a young man today to successfully terminate his studies in medicine, he must have that same ability and that same tenacity of purpose that is so necessary not only for the student but for the practitioner of medicine. Today, if the faculty find that a student exhibits a lukewarmness or inaptitude for his work he is courteously recommended to discontinue the study of medicine. Should he not act upon this suggestion, the faculty may at their discretion refuse further matriculation, or the sifting process of the examinations may leave him high and dry. The public is thus saved from the young man and, what is still more important, the young man is saved from himself.

Such methods, however, are very rarely necessary, as the large majority of our students are men and women of earnest purpose and ability. Now, if the foregoing be all wrong as to motives for your adopting the study of medicine, what then should be the true reason for your undertaking the work? It should be primarily your love for science in general and your desire for medicine in particular. With this should be coupled a thorough realisation of the arduous duties and the grave responsibilities that will be yours in your life of administering to the sick; with this high incentive and, of course, the requisite ability you may confidently expect success,—success not only in your college work but in your later professional career.

Success, though, is a relative term. Each of us has his own conception of what the word comprehends, depending upon his own individual ideas. But success in your college work must mean to you all one thing at least,—namely, that you pass the examinations. This, however, is the very lowest possible standard that you can have for your student work. Success to you as students should mean something very much more. It should mean that when you leave this college you should possess a training that will enable you to successfully cope with medical problems unhinted at in your student days; it should mean a training that will stimulate you to seek and possibly find new paths in your profession; it should mean that training which broadens your horizon to things other than those in the science of medicine itself; and, finally, it should mean a training that will inspire and prompt the best and the noblest that is in you, not only in your profession but in your lives as men. If at the termination of your studies you can claim these, then indeed have you attained success.

After you have entered the practice of your profession your individual ideas of success will vary within very wide limits. To one it may mean simply the acquiring of a snug bank account; to another it may mean a modest country practice; to still another it may mean high standing in the ranks of your profession; and so each will have his own notion of what spells success, and toward that ideal it is to be hoped that he will strive. It is not my purpose, therefore, to attempt to define for you what success in your professional life means, for it would be my idea of success and perhaps not yours at all, but I think that I may be permitted to make a few suggestions which, if followed, will tend to lead to success in your student work, as well as to success in your professional life, no matter what your idea of success may be.

I like to compare these opening lectures with the ceremonies and first shovelful of earth that inaugurates the beginning of work upon a great public building. Tonight we start the work for you and tomorrow you are to take up your pick and shovel and are to labor for four long years, in the construction of a foundation upon which in later years you are to erect a building as beautiful and large as you will. Much, or perhaps all, depends upon the foundation you fabricate. It must be such that you go on and on with a structure that should never be completed; for the moment you cease building, that moment your building becomes old and decay sets in. It is not, however, of this building that I wish to speak, for we feel sure that if your foundation is right the building will take care of itself. If your four years of apprenticeship have been properly employed here, you will emerge as master workmen, prepared and anxious to go on. And so now as students we want you to thus realise that you are here not so much for the purpose of studying to learn medicine, but rather learning to study medicine. Keep clearly this thought before your mind; its truth you will better realise when you have left this institution.

Now then, what is necessary that you may properly construct this foundation which is to be the base of your work in later years? Your professors and instructors will supply abundant material, much more I fear than you will ever use; but just here remember that good work depends not so much upon the amount of material you use, but upon how well you use it. Each stone should be carefully fitted in its relations to those about it. Loose material lying about is of little value. No matter how good in itself a stone may be, it is of no true service to you until you have made it a part of the whole. In this same way, in your college work never allow a fact to remain loose and unattached. As such it can be of no real value. Find some other fact which depends upon it or some fact upon which it depends, cement these together with your best reasoning, and then it is fixed and becomes yours forever.

If I were asked for the one thing that will contribute to the best training in your student work, I should unhesitatingly reply, "the exercise and development of your faculties in reasoning from the cause to the effect in medicine,"—the "why and what of it" in medicine, if you like. It is to the development of this that your professors and instructors try to lead you and it is to this end that you yourselves must strive. Be not satisfied with an isolated fact or statement, but fit it to some other by the answer to one or both questions, "why and what of it?" The cultivation of this habit will make you masters of what you learn and you will be thus able to draw upon your knowledge to good purpose, in the solution of those everyday problems that will confront you in your professional life. Some one has said that as important as having knowledge is the knowing how to use it, and this habit of correlating one subject with another will help to fix all, make them equally useful and train you to use them to the best advantage. This spirit of inquiry developed during your work here will prompt you to investigation and research, to solve questions in medicine as yet unanswered, and thus perhaps you will bring fame to yourself and inestimable good to others.

Next in importance to the proper use of material comes the enthusiasm with which you work. In building this foundation we want no union men. Yours is not to be simply an eight hour day but it may be a ten or twelve. Your zeal for work, however, will not be wanting if your motive in studying medicine has been the proper one. Perhaps you remember Richard Carston in Dickens's "Bleak House," who, after trying several different professions and occupations and giving them all up from lack of interest and intensity of purpose, was finally sent to a kind surgeon, who undertook to instruct him in his profession. This, too, poor Richard in a short time gave up, and upon his friend's enquiring of the surgeon's wife as to the cause of the failure, she replied that it was a favorite saying of the captain who was the predecessor of the surgeon in the love of the good woman, "that when you make pitch hot, you cannot make it too hot, and if it is only a plank you have to swab, swab it as if Davy Jones were after you," and she concluded by remarking that she thought this applied to medicine as well as to other pursuits.

There are very few of us who can work with enthusiasm along lines that do not appeal to us and arouse our interest. If we are not interested in the work we have in hand it is a difficult matter to keep our "pitch hot." Some very fortunate ones are able to put their best efforts in whatever work they do, regardless of whether it appeals to their interest or not. These are men who attain success in whatever they undertake, whether profession or business. It is a rare attribute and a valuable one for its possessor. The opposite of these is typified in Richard Carston, to whom nothing appealed. Men of such stamp drift along aimlessly through life, never attaining success nor leaving the imprint of their personality upon what they do. Most of us, however, belong to that great majority who require the stimulus and prompting of congenial work. Our work must possess for us a continual unabated interest in order that we may do the best that is in us. And this, then, is an important reason for you to scrutinise carefully tonight your motives in studying this profession, and should you even later find that medicine does not meet your requirements, by all means abandon it for pursuits more adapted to your capabilities and tastes, and in which you will be able to do greater justice to yourself, as well as be of greater use to your fellow men.

Thus far my remarks have been in your interests only, but I do not want the opportunity to pass without saying a word in behalf of your instructors. Bear in mind that you teachers have always your welfare and advancement at heart. You can show your appreciation by giving your attention, for an instructor is always grateful for the interest you manifest in his subject. Over the piano in a western dance hall was displayed a sign reading, "Please don't shoot the band; he is doing his best." I have sometimes thought that a sign in your recitation room asking you not to discourage your teacher, as he is doing his best, might not be out of place. It is very discouraging for the instructor to teach and to supply the enthusiasm that the student should bring with him. An attempt at answer will always be appreciated for it indicates a desire on your part to try at least. And remember in this connection, that your reputation as students is built not upon what you know but upon what we find out you know.

Just here let me say that it is unfortunate that we must depend to such a great extent upon the examination as a criterion of your knowledge. Theoretically, the examination is right; practically, it is wrong. It is wrong because the student makes the examination his goal, and the moment he does he loses sight of that true significance of his medical training which I have tried to impress upon you tonight. Just so long as the student regards the examination in this light, just so long will he appear at the time of the examination haggard and worn, the result of an attempt to crowd the subject matter of a year into the space of a few hours. And if on the night of the examination he were given his choice between a mark of eighty and a high ideal, he would naturally select the mark. We set a premium on the mark and not on the ideal. To attain that mark he has crowded into a tired brain a jumbled mass of facts of absolutely no use to him, except for the purposes of the examination and then they are as promptly forgotten. The result is that in these cases we turn out a well crammed graduate and not a thinker.

Look at the question as we will we can not get away from the fact that we as your teachers are primarily responsible for this state of affairs. But the examination with all its evils must stand for the present, until some better standard may be had to indicate your fitness for advancement. Until that time shall come we both must recognise its evil influence, and you as students and we as teachers, must work together for the accomplishment of that broader significance of medical training. Efforts in that direction have been made by teachers of medicine all over the world, and the recitation, the conference, and the more general bedside teaching are steps in the right direction. Your parts as students will come when you better realise the importance of doing your work each day so that it will play its part in shaping your character and adding permanent knowledge to your store, so that you may truly say,

"Each day is your life,
"And your life but a day repeated."

And now in closing I am conscious that I have not been able to give you anything that is either scholarly or scientific. But I shall be content if I have succeeded in implanting one thought that will help you to broaden your scope and view of your work with use; one thought that will help you to realise the importance of your work here as not meaning simply the obtaining of a degree, but the acquiring of a training that will develop in each of you a scholar. And so when you shall have received from our hand the highest honor which lies in our power to bestow, we shall not feel that you are "well crammed graduates," but well rounded scholars of medicine.

With this hope goes my God speed and the assurance that the entire faculty is with you in your work, with desire to assist you in developing that student ideal, which will make men and women of worth, physicians of attainment, each a credit to this University.

1248 MAIN STREET.

